# 

**Published:** 1910-07-15

**Authors:** 


### ANNOUNCEMENTS.

Northern Ohio Dental Association.—The fifty-third annual meeting of the Northern Ohio Association was held in the Secor Hotel at Toledo, June 7th, 8th and 9th. The sessions were largely attended and the programme was one one of unusual excellence. Six excellent papers were read and excellently discussed and about forty clinics were given. This is the second oldest society in existence. But though old it is strong and active and promises to live and flourish for many years more. Dr. W. G. Ebersole of Cleveland was elected President

---

MICHIGAN STATE DENTAL SOCIETY.—The fifty-fourth annual meeting of this society was held in the Fuller Hotel at Detroit June 13th, 14th and 15th. The meeting was largely attended and because of the strong program and excellent management the meeting was full of valuable and interesting features. The closing incident of the meeting was a boat trip down the river and back. The weather was ideal and the Detroit dentists who supplied the entertainment are to be congratulated and commended for so generous hospitality. Dr. D. M. Graham of Detroit was elected President.

---

AMERICAN MILLER MEMORIAL. Honor to whom honor is due. All dentists at home and abroad are debtors to the late W. D. Miller, whose scientific mind, untiring energy and pride in American citizenship has been one of the potent factors in placing this country at the head of the profession. Let the old adage "A prophet is not without honor save in his own country" be the exception in this instance and every loyal American be represented in a lasting memorial to his useful life.

The proposed bronze will be erected in the capital city of his native state, that he may share the well-merited distinction of being one of our greatest men.

The invitation to contribute is extended individually, and collectively, through the societies of the country and your support, both moral and financial, is urgently solicited.

In order that the work may be quickly and efficiently accomplished, contributions may be sent direct to the Treasurer of the American Miller Memorial Fund: Weston A. Price, 10406 Euclid Ave., Cleveland, Ohio. Executive Committee,

EDWARD C. MILLS, Columbus, O.
J. R. CALLAHAN, Cincinnati, O.
S. D. RUGGLES, Portsmouth, O.

International Hygiene Exhibition, Dresden, 1911. —*Appeal.*—A grand *International Hygiene Exhibition* will be held in *Dresden* from May to October next year, under the immediate patronage of the King of Saxony. Among the many groups, which are to include all branches of Hygiene, will be the special group "Diseases of the Teeth." The undersigned are entrusted by the Directorium of the Exhibition with the organization of the *scientific* portion of this group. In accordance with the plans of the Directorium the causes, the distribution, the prevention, and the treatment of diseases of the teeth will be given the first place in our scheme of arrangement, with special regard to social circles. We therefore appeal to our professional colleagues throughout the world to send us objects suitable for the *scientific* portion of this group, so as to enable us to present as complete and uniform a view as possible of the diseases of the teeth and of modern achievements in dental hygiene.

The "Federation Dentaire Internationale" has already promised its extremely valuable assistance. As intermediaries will act: the General Secretary of the F. D. I., Dr. Schaffer-Stuckert, of Frankfort o. M., for the National Committee of the Federation, and Professor Dr. Jessen, of Strasburg, for the National Committee of the H. C. F. D. I. As for Germany, Professor Dr. Dieck of Berlin, has undertaken to communicate with exhibitors.

The following programme has been published:

I. Causes of tooth decay.
   Micro-organisms.
   Influence of mode of life and nourishment.
   Relation to general diseases of the body.
   Heredity.

II. Distribution of tooth diseases.
   According to age, sex, occupation.
   Significance of unsound teeth for health of nation.

III. Prophylaxis of tooth diseases.

IV. Prevention of tooth diseases.
   In the school.
   In the army.

In factories and in institutions for the common welfare.
Through state insurance.

*Objects for the Exhibition should be notified to the undersigned, if possible, not later than the 1 July 1910, and sent to Dresden so as to arrive by the end of February 1911.*

The Chairman of the sub-section "Care of Youth" has requested our group to supply him with *duplicates* of our exhibits—if any are available and so far as they come within the province of dental clinics for schools—for exhibition in his sub-section. Professor *Walkhoff* is prepared to communicate to the Directorium of the Exhibition all information concerning such duplicates for that (Care of Youth) or any other subsection or group; as well as all questions of a general nature.

Forms of notification may be obtained direct from the offices of the International Hygiene Exhibition, Dresden, Zwickauerstr. 35, and when filled up, should be addressed to the undersigned—except from places in Germany, whence they should be sent only to *Professor Dr. Dieck, Berlin, Potsdamerstrasse 113.*

The fullest possible cooperation on the part of the profession in all countries, as at the last International Congress in Berlin, will be of the greatest use, not only to the cause of our Exhibition, but to the whole profession of Surgeon Dentists. We desire to show the world, that our profession, when judged by the exhibitions in other subsections or groups, is second to none in the domain of hygiene.

Prof. *Dr.* Med. WALKHOFF, Munchen, President.
Prof. *Dr.* Med .DIECK, Berlin Prof. *Dr.* Med. JESSEN, Strasburg · Dr. SCHAFFER-STUCKER Frankfort o. M., Vice-Presidents.

